# Mitomycin C, vinblastine and cis-platin. An active regimen for advanced non-small cell lung cancer.

**DOI:** 10.1038/bjc.1987.227

**Published:** 1987-10

**Authors:** G. Giaccone, M. Bagatella, M. Donadio, G. M. Bonardi, F. Testore, P. Ferrati, L. Ciuffreda, A. Calciati

**Affiliations:** Division of Medical Oncology, Ospedale S. Giovanni, Torino, Italy.

## Abstract

Fifty-one patients with advanced non-small cell lung carcinoma were treated with a combination of mitomycin C, vinblastine and cis-platin (MVP). Most cycles were given on an out-patient basis. Major side effects were leukopenia and peripheral neurotoxicity; one patient died of sepsis while leukopenic. In 44 evaluable patients the response rate was 50%, with one complete response. Overall median survival time was 280 days and median duration of responses was 232 days. A better performance status, disease limited to one hemithorax and no prior exposure to chemotherapy positively influenced the survival. MVP is an effective chemotherapy for non-small cell lung cancer and further experience with this combination is warranted.


*Br. J. Cancer* (1987), **56**, 475–478

© The Macmillan Press Ltd., 1987

### Mitomycin C, vinblastine and *cis*-platin. An active regimen for advanced non-small cell lung cancer

G. Giaccone[1], M. Bagatella[1], M. Donadio[1], G.M. Bonardi[1], F. Testore[2], P. Ferrati[1], L. Ciuffreda[1] & A. Calciati[1]

[1]*Division of Medical Oncology, Ospedale S. Giovanni, Antica Sede, Torino; and* [2]*Division of Internal Medicine, Ospedale Civile, Asti, Italy.*

**Summary** Fifty-one patients with advanced non-small cell lung carcinoma were treated with a combination of mitomycin C, vinblastine and *cis*-platin (MVP). Most cycles were given on an out-patient basis. Major side effects were leukopenia and peripheral neurotoxicity; one patient died of sepsis while leukopenic. In 44 evaluable patients the response rate was 50%, with one complete response. Overall median survival time was 280 days and median duration of responses was 232 days. A better performance status, disease limited to one hemithorax and no prior exposure to chemotherapy positively influenced the survival.

MVP is an effective chemotherapy for non-small cell lung cancer and further experience with this combination is warranted.

Chemotherapy has a limited activity in non-small cell lung carcinoma (NSCLC); single agents achieve around 20% response rate, whilst combination chemotherapy containing *cis*-platin (DDP) may obtain up to 60% response rate (Sculier & Klastersky, 1984; Bakowski & Crouch, 1983). Although multiple drug regimens seem to obtain higher response rates than single agents, their toxicity is often remarkable, especially when DDP is one of the drugs (Ruckdeschel *et al.*, 1986); moreover, complete remission rate still remains below 10% even with the most aggressive regimens, and, finally, no survival advantage has so far been demonstrated in comparison with a no-treatment arm (Woods *et al.*, 1985). Thereby, chemotherapy should still be regarded as investigational and not routine treatment in NSCLC. The best known and frequently employed regimens in NSCLC contain DDP with either a vinca alkaloid (vindesine or vinblastine) or etoposide (VP16 213) (Ruckdeschel *et al.*, 1986; Gralla *et al.*, 1981; Longeval & Klastersky, 1982). Mitomycin C has shown efficacy in the treatment of NSCLC, with a response rate of about 20% (Bakowski & Crouch, 1983; Samson *et al.*, 1979).

In the attempt to increase response rate and eventually improve survival, we treated patients with NSCLC with an aggressive combination of high-dose DDP, vinblastine and mitomycin C.

### Materials and methods

Fifty-one patients with histologically or cytologically documented advanced NSCLC were entered in the study from September 1984 to June 1986 (Table I).

No patient was amenable to curative surgery or radiation, or had a performance status (ECOG) >3. Measurable or evaluable disease was required of each subject. Bone lytic lesions were not considered evaluable if they were the only sites of disease. Prior chemotherapy was permitted, as well as radiation to lesions not used for response assessment. Patients were required to be off prior treatment for a minimum of 3 weeks and any toxicity associated with prior therapy resolved before entry into the study. Patients had to be no more than 70 years of age, have normal renal function (serum creatinine $<1.5\,mg\,dl^{-1}$ and/or creatinine clearance $>60\,ml\,min^{-1}$), normal marrow (leukocytes $\geq 4{,}000\,mm^{-3}$, platelets $\geq 100{,}000\,mm^{-3}$), normal liver function (bilirubin $<1.5\,mg\,dl^{-1}$) and normal cardiac function. Life expectancy was required to be at least 2 months. Informed consent was required from all patients.

Patients were administered: Mitomycin C (MMC) $10\,mg\,m^{-2}$ on days 1, 57 and then every 12 weeks, vinblastine (VBL) $5\,mg\,m^{-2}$ on days 1, 8, 15, 22, 29 and then every 2 weeks, DDP $100\,mg\,m^{-2}$ on days, 1, 29, 57 and then every 6 weeks. Up to six DDP and two further VBL doses were given to responding and stable patients, for a total of 30 weeks of treatment (Table II); in the early phase of the study a responding patient erroneously received 7 DDP cycles. Dose modifications were applied to the administration of VBL when given alone according to the following: if leukocytes (WBC) $2\text{–}3{,}000\,mm^{-3}$ and/or platelets $75\text{–}100{,}000\,mm^{-3}$ a 50% dose was given; if WBC $<2{,}000$ and/or platelets $<75{,}000$ the drug was not given.

VBL and DDP, with or without MMC were given only if WBC and platelets counts were $>4{,}000$ and $>100{,}000$ respectively; patients requiring more than 2 weeks delay were withdrawn from therapy.

DDP was administered only if serum creatinine was $<1.5\,mg\,dl^{-1}$. Hydration comprised 2.5 l fluids and forced mannitol diuresis. Infusion lasted 4–6 h overall and most cycles were given in an out-patient setting. If during treatment creatinine increased up to $1.5\text{–}2.0\,mg\,dl^{-1}$, a 24 h hydration was applied after normalization of the creatinine level. If creatinine increased up to $2.0\text{–}3.0\,mg\,dl^{-1}$, a 50% DDP dose was administered, with a 24 h hydration. If during treatment creatinine increased above $3.0\,mg\,dl^{-1}$, DDP was withdrawn and treatment continued with 100%VBL and 50% MMC.

VBL and DDP doses were reduced by 50%, if occurrence of sensory peripheral neurotoxicity prevented patient's normal activities. If severe motor neurotoxicity or ileus occurred chemotherapy was discontinued.

Patients were classified as having limited disease if tumour was confined within one hemithorax and regional lymphatics, including ipsilateral supraclavicular lymphnodes and ipsilateral pleural effusion. Extensive disease was defined as that beyond the limits mentioned above.

Chest X-ray and imaging of marker lesions were performed before commencement of chemotherapy and before every cycle of DDP together with standard biochemical analysis of the blood. Haematological counts were repeated before every drug administration.

Response criteria and toxicity grading were those recommended by the WHO (1979). Response duration and survival time were computed from the start of treatment. Actuarial survival was estimated by the method of Kaplan & Meier (1958), and differences between survival curves were computed by the log-rank test (Mantel, 1966).

Correspondence: G. Giaccone.
Received 19 May 1987; and in revised form, 23 June 1987.

**Table I** Patient characteristics

| | |
|---|---|
| Total number/evaluable patients | 51/44 |
| Sex: male/female | 46/ 5 |
| Age: median (range) | 57 (35–71) |
| Performance status (ECOG): 0–1 | 33 |
| 2–3 | 18 |
| Histology: squamous | 21 |
| adeno | 17 |
| large cell | 9 |
| others | 4 |
| Weight loss: ≦10%/>10% | 37/14 |
| Stage: limited/extensive | 13/38 |
| Prior treatment: none | 39 |
| chemotherapy | 8 |
| radiotherapy | 8 |
| radiotherapy + chemotherapy | 4 |
| surgery | 7 |

### Results

Forty-four of 51 patients were evaluable for response; 5 patients died within 4 weeks from the start of chemotherapy (4 died of disease and 1 died of sepsis while severely leukopenic), 1 patient refused further treatment after day 1 due to intense nausea and vomiting, and 1 patient had no evaluable lesions. Thirty-eight patients had extensive disease; metastatic sites were as follows: 15 bone, 13 lymph nodes, 10 lung, 4 liver, 4 adrenal, 3 skin, 2 central nervous system, 1 pericardium and 1 pleura.

Thirty-nine patients were previously untreated by any modality.

All patients have so far completed chemotherapy, and 204 cycles of DDP have been administered overall (a median of 4 cycles per patient; range 1–7).

The main toxicity has been marrow toxicity (Table III). One patient died from sepsis on day 13 of the first cycle, while WBC counts were 200 $mm^{-3}$. Leukopenia and thrombocytopenia of grade 3 and 4 occurred in 45% and 4% of patients, respectively; anaemia was observed in 79% of patients overall and was severe in 2.

Forty-seven patients required either VBL dose reduction or omission at least once, mostly due to leukopenia. DDP delay was required in 21 patients, mainly due to marrow toxicity; however, in no case did myelotoxicity cause a delay in chemotherapy of more than 2 weeks.

Nausea and vomiting were distressing in 67% of patients, despite prophylactic antiemetic medications (intermediate dose metoclopramide plus dexamethasone). Transient and reversible nephrotoxicity was seen in 7 patients (1 patient discontinued DDP due to elevation of serum creatinine up to 3.4 $mg\,dl^{-1}$). One patient had severe hypokalaemia (2.1 $mEq\,l^{-1}$). Peripheral neurotoxicity was moderate (WHO grade 2) in 3 patients and severe (WHO grade 3) in 3 other patients. Constipation occurred in 15 patients and was troublesome in 2 patients (ileus in 1 patient). Symptomatic ototoxicity was encountered in 4 patients. Diarrhoea occurred in 14 patients, mucositis in 5, fever in 4, skin allergy in 2 and severe infection in 3 patients (septic death in 1). Phlebitis occurred in 5 cases; extravasation of VBL occurred in 2 cases but did not require skin grafting.

**Table III** Toxicity (percent of patients; Highest grade recorded)

| *Grade (WHO)* | *0* | *1* | *2* | *3* | *4* |
|---|---|---|---|---|---|
| Leukopenia | 8 | 14 | 33 | 35 | 10 |
| Thrombopenia | 88 | 4 | 4 | 4 | |
| Anaemia | 21 | 41 | 34 | 2 | 2 |
| Emesis | 6 | 2 | 25 | 45 | 22 |
| Diarrhoea | 72 | 14 | 14 | | |
| Mucositis | 90 | 6 | 4 | | |
| Hepatic | 100 | | | | |
| Pulmonary | 100 | | | | |
| Renal | 86 | 12 | 2 | | |
| Haematuria | 96 | 4 | | | |
| Cardiac | 100 | | | | |
| Neurological | 68 | 20 | 6 | 6 | |
| Constipation | 70 | 16 | 10 | 4 | |
| Fever | 92 | 2 | 6 | | |
| Skin allergy | 96 | 4 | | | |
| Infection | 72 | 14 | 8 | 4 | 2 |
| Alopecia | 48 | 25 | 25 | 2 | |
| Others[a] | 59 | 37 | 2 | 2 | |

[a]Gastric pain, phlebitis, asthaenia, drug extravasation, hypokalaemia, tinnitus.

Stomach ache was recorded in 8 patients. Some hair loss was observed in 25 patients, but was complete in only one case.

Among 44 evaluable patients there were one complete response (confirmed by bronchoscopy and thorax CT scan), 21 partial responses (total response rate 50%), 15 no change (NC), and 7 progressions (PD). If the 4 patients who died from disease within the first 4 weeks of treatment are considered as PD, the response rate becomes 46% (22/48). Fifty percent of patients with and without prior exposure to chemotherapy responded to MVP (3/6 and 19/38 evaluable patients, respectively); if early deaths are taken into account 37% (3/8) and 48% (19/40) responded to MVP, respectively. Median duration of responses was 232 days; the patient in complete response continues 694 days from the start of chemotherapy and 443 days from its termination. The median duration in the NC patients is 222 days. Actuarial median follow-up is 361 days (10–640 days). Median survival time (MST) was 280 days. Eighteen patients are still alive (Figure 1). Table IV shows median survival times in relation to major prognostic factors.

**Table IV** Median survival time

| | *Number of patients* | *Median survival time (days)* |
|---|---|---|
| Overall | 51 | 280 |
| Performance status 0–1 | 33 | 458 |
| 2–3 | 18 | 189 |
| Disease extent: limited | 13 | 458 |
| extensive | 38 | 239 |
| Prior chemotherapy: no | 43 | 298 |
| yes | 8 | 203 |
| Responders (CR + PR) | 22 | 351 |
| Non-responders (NC + PD) | 22 | 223 |
| Patients with NC | 15 | 280 |
| Weight loss: >10% | 14 | 268 |
| ≦10% | 37 | 298 |

**Table II** MVP schedule

| | | | | | | | | | | | | | | | | | | |
|---|---|---|---|---|---|---|---|---|---|---|---|---|---|---|---|---|---|---|
| MMC | X | | | | | | X | | | | | | X | | | | | |
| VBL | X | X | X | X | X | X | X | X | X | X | X | X | X | X | X | X | X | X |
| DDP | X | | | | X | | X | | | X | | | X | | | X | | |
| Week | 0 | 1 | 2 | 3 | 4 | 6 | 8 | 10 | 12 | 14 | 16 | 18 | 20 | 22 | 24 | 26 | 28 | 30 |

MMC = mitomycin C 10 $mg\,m^{-2}$; VBL = vinblastine 5 $mg\,m^{-2}$; DDP = *cis*-platin 100 $mg\,m^{-2}$.

**Figure 1** Overall survival curve

A significant difference in survival was apparent between patients with limited and extensive disease ($P<0.025$). Performance status was an important prognostic factor: patients with PS 0 or 1 survived significantly longer than those with PS 2 or 3 ($P<0.001$).

Prior exposure to chemotherapy also influenced survival, though the number of pretreated patients is small ($P<0.01$). Also, response to treatment was an important prognostic factor: Responding patients survived a median of 351 days in comparison to those who had NC or PD (223 days; $P<0.025$). The difference was even more striking when responders were compared to progressing patients (MST 351 *vs.* 151 days; $P<0.001$). No significant difference existed in survival between responders and NC ($P>0.1$). Although the survival advantage of responders over non-responders does not mean that responsive patients survived longer because of the treatment, patients who responded to chemotherapy might represent a category of people who, for other reasons have a longer survival.

Eight patients have survived more than one year, and among these 5 had responded, 2 had NC and one was not evaluable for response (response rate 71%).

Weight loss in the last 3 months did not apparently influence survival ($P>0.1$).

### Discussion

The most frequently used and effective combinations in NSCLC contain DDP and a vinca alkaloid (either vindesine or vinblastine) or VP16.313.

The addition of a third drug to these regimens has been attempted by several investigators with the aim of increasing response rate and possibly survival. Mitomycin C is known as one of the active agents in NSCLC (Bakowski & Crouch, 1983; Samson *et al.*, 1979); its addition to the DDP–vindesine regimen significantly increased the response rate from 27% to 54% in the preliminary report by Gralla *et al.* (1986) on 120 patients.

On the other hand, a 23–59% response rate has been reported by several authors, with vindesine and mitomycin alone (Luedke *et al.*, 1986; Main *et al.*, 1986; Sculier *et al.*, 1986).

However, the overall advantage of a three drug regimen over a two drug conbination has still to be demonstrated, especially if we consider the higher toxicity caused by addition of a third drug (namely DDP).

The large ECOG trial comparing the four most active regimens for metastatic NSCLC (CAMP, DDP–vindesine, DDP–VP16.213, MVP), failed to demonstrate an advantage in survival of any arm over the others; nevertheless, the MVP schedule, which employed a lower DDP dose than ours ($40\,mg\,m^{-2}$) obtained a response rate (31%) which was significantly higher than that of the other regimens in patients with squamous carcinoma and adenocarcinoma. Overall, toxicity was noticeable in all DDP-containing regimens, but in particular nephrotoxicity was more frequent and severe in the DDP–vindesine arm (Ruckdeschel *et al.*, 1986).

In our study the addition of mitomycin C to DDP and vinblastine has apparently improved our previous experience in treating advanced NSCLC, over a DDP–VP16.213 combination in a similar group of patients (Giaccone *et al.*, 1984), although the comparison is not randomized.

The 50% response rate obtained in our trial with MVP is similar to that reported by other groups (Schulman *et al.*, 1983; Mason & Catalano, 1980); although we obtained only one complete remission.

Emesis was as frequent and intense as we expected, despite prophylactic antiemetic treatment; neurotoxicity became a problem in a significant proportion of treated patients; in fact, 6 and 7 patients had moderate to severe peripheral neurotoxicity and constipation, respectively. Myelotoxicity has been universal, but an appropriate dose modification schedule applied to vinblastine administration and a reduction of intensity of chemotherapy after the two initial 4-weekly courses contained the life-threatening episodes; nevertheless one patient eventually died from sepsis while severely leukopaenic during the first cycle.

Our study confirmed performance status, disease extent and prior chemotherapy exposure as prognostic factors in NSCLC. In conclusion, the MVP combination is active in NSCLC, although the complete response rate is still unsatisfactory in the treatment of this disease. A careful evaluation of aggressive therapies like MVP has to be performed; further studies are warranted, especially in combined modality trials, as in a neoadjuvant setting, in borderline operable patients.

### References

BAKOWSKI, M.T. & CROUCH, J.C. (1983). Chemotherapy of non-small cell lung cancer: a reappraisal and a look to the future. *Cancer Treat. Rev.*, **10**, 159.

GIACCONE, G., FERRATI, P., DONADIO, M. & 4 others (1984). Chemioterapia d'associazione con *cis*-Platino e VP 16-213 nei carcinomi del polmone inoperabili non-microcitomi. *Acta Oncol.*, **5**, 187.

GRALLA, R.J., CASPER, E.S., KELSEN, D.P. & 5 others (1981). *Cis*-platin and vindesine combination chemotherapy for advanced carcinoma of the lung: A randomized trial investigating two dosage schedules. *Ann. Int. Med.*, **95**, 414.

GRALLA, R.J., KRIS, M.G., BURKE, M.T., KELSEN, D.P. & HEELAN, R. (1986). The influence of the addition of mitomycin (M) to vindesine (V) plus *cis*-platin (P) in a random-assignment trial in 120 patients with non small cell lung cancer (NSCLC). *Proc. Am. Soc. Clin. Oncol.*, **5**, 182 (*abstract*).

KAPLAN, E., & MEIER, P. (1958). Nonparametric estimation from incomplete observations. *J. Am. Statist. Assoc.*, **53**, 457.

LONGEVAL, E. & KLASTERSKY, J. (1982). Combination chemotherapy with *cis*-platin and etoposide in bronchogenic squamous cell carcinoma and adenocarcinoma: A study by the EORTC Lung Cancer Working Party (Belgium). *Cancer*, **50**, 2751.

LUEDKE, D.W., LEUDKE, S.L., MARTELO, O. & 5 others (1986). Vindesine and mitomycin in the treatment of advanced non-small cell lung cancer: A Southeastern Cancer Study Group trial. *Cancer Treat. Rep.*, **70**, 651.

MAIN, J., CLARK, R.A. & HUTCHEON, A. (1986). Vindesine and mitomycin C in inoperable non-small cell lung cancer. *Eur. J. Cancer Clin. Oncol.*, **22**, 983.

MANTEL, N. (1966). Evaluation of survival data and two new rank order statistics arising in its consideration. *Cancer Chemother. Rep.*, **50**, 163.

MASON, B.A. & CATALANO, R.B. (1980). Mitomycin, vinblastine and *cis*-platin combination chemotherapy in non-small cell lung cancer. *Proc. Am. Soc. Clin. Oncol.*, **21**, 447 (*abstract*).

RUCKDESCHEL, J.C., FINKELSTEIN, D.M., ETTINGER, D.S. & 4 others (1986). A randomized trial of the four most active regimens for metastatic non-small cell lung cancer. *J. Clin. Oncol.*, **4**, 14.

SAMSON, M.K., FRAILE, R.J., LEICHMAN, L.P. & 4 others (1979). Clinical studies of mitomycin C in advanced adenocarcinoma of the lung. In *Mitomycin C: current status and new developments*, Carter, S.K. & Crooke, S.T. (eds) p. 121. Academic Press: New York.

SCHULMAN, P., BUDMAN, D.R., WEISELBERG, L., VINCIGUERRA, V. & DEGMAN, T.J. (1983). Phase II trial of mitomycin, vinblastine, and *cis*-platin (MVP) in non-small cell bronchogenic carcinoma. *Cancer Treat.Rep.*, **67**, 943.

SCULIER, J.P. & KLASTERSKY, J. (1984). Progress in chemotherapy of non-small cell lung cancer. *Eur. J. Cancer Clin. Oncol.*, **20**, 1329.

SCULIER, J.P., KLASTERSKY, J.P., DUMONT, G. & 7 others (1986). Combination chemotherapy with mitomycin and vindesine in advanced non-small cell lung cancer: A pilot study by the Lung Cancer Working Party (Belgium). *Cancer Treat. Rep.*, **70**, 773.

WHO (1979). *Handbook for reporting results of cancer treatment*. WHO offset Publ. no. 48. WHO: Geneva.

WOODS, R.L., LEVI, J.A., PAGE, J. & 4 others (1985). Non-small cell lung cancer: A randomized comparison of chemotherapy with no chemotherapy. *Proc. Am. Soc. Clin. Oncol.*, **4**, 177 (*abstract*).


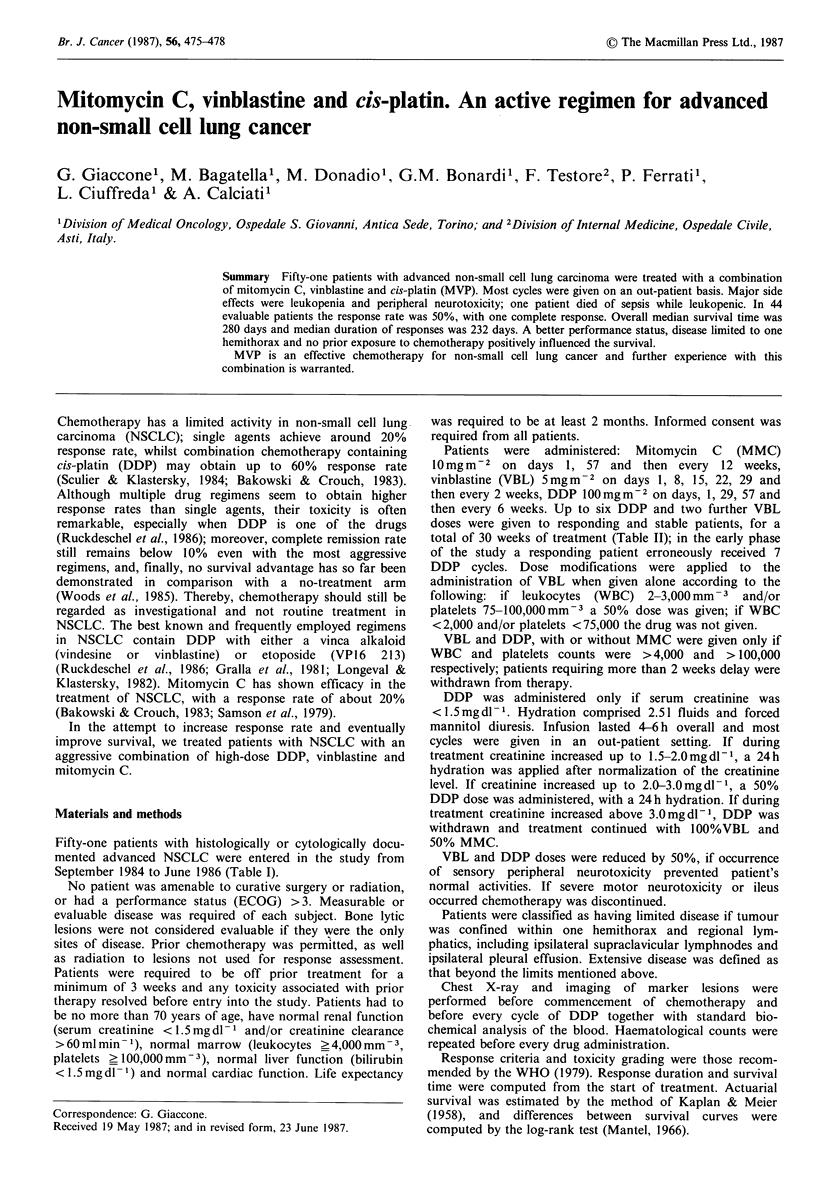

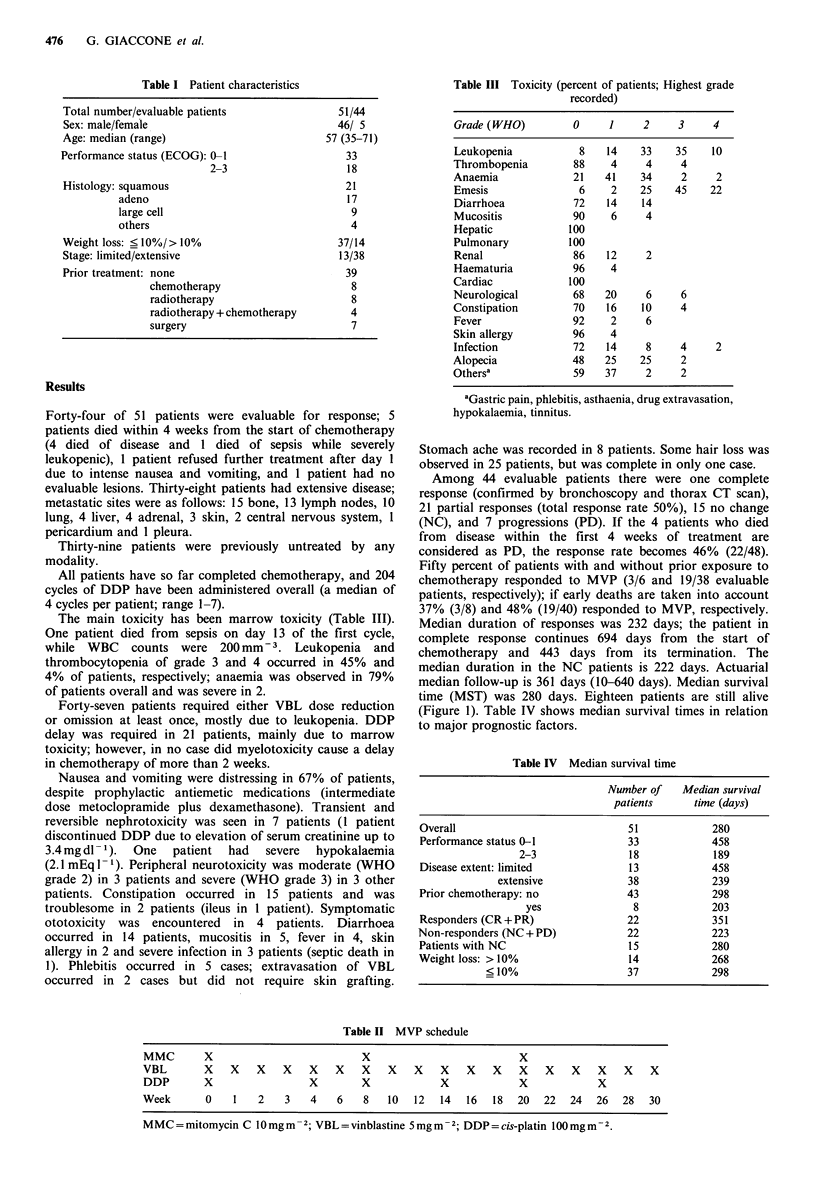

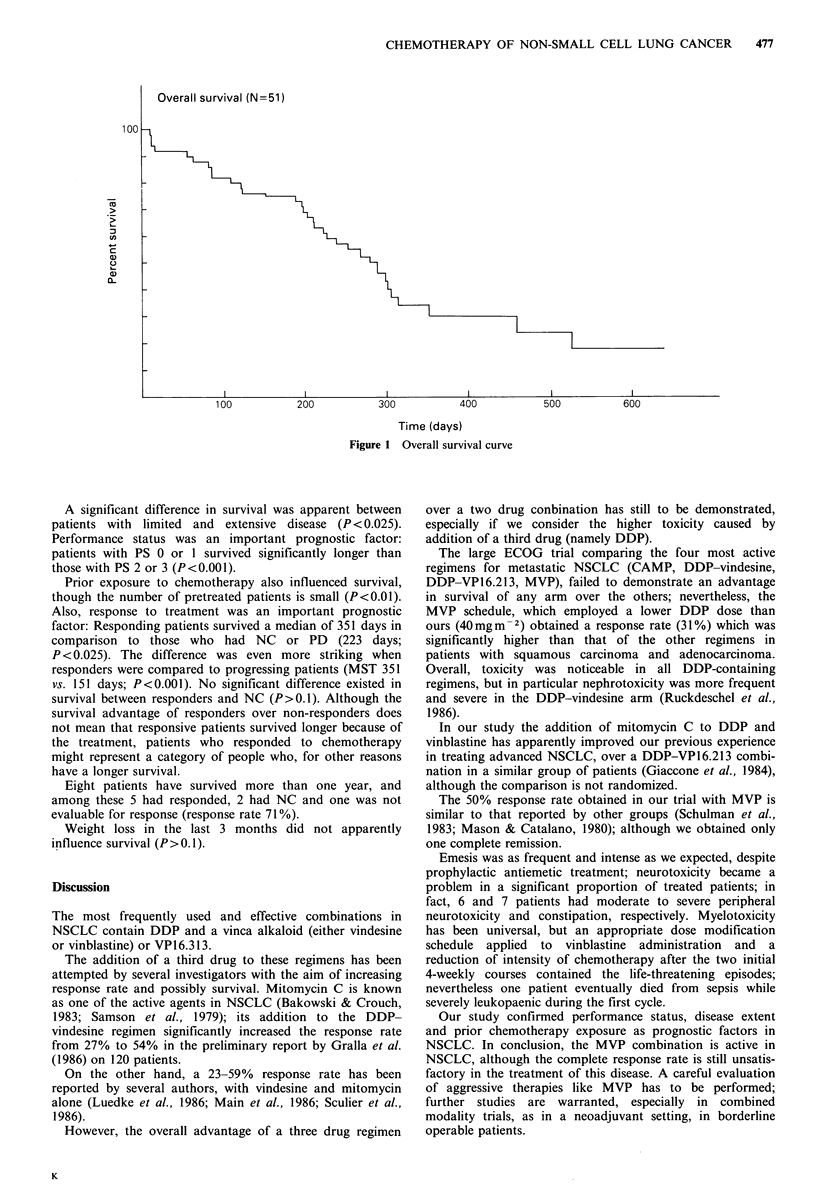

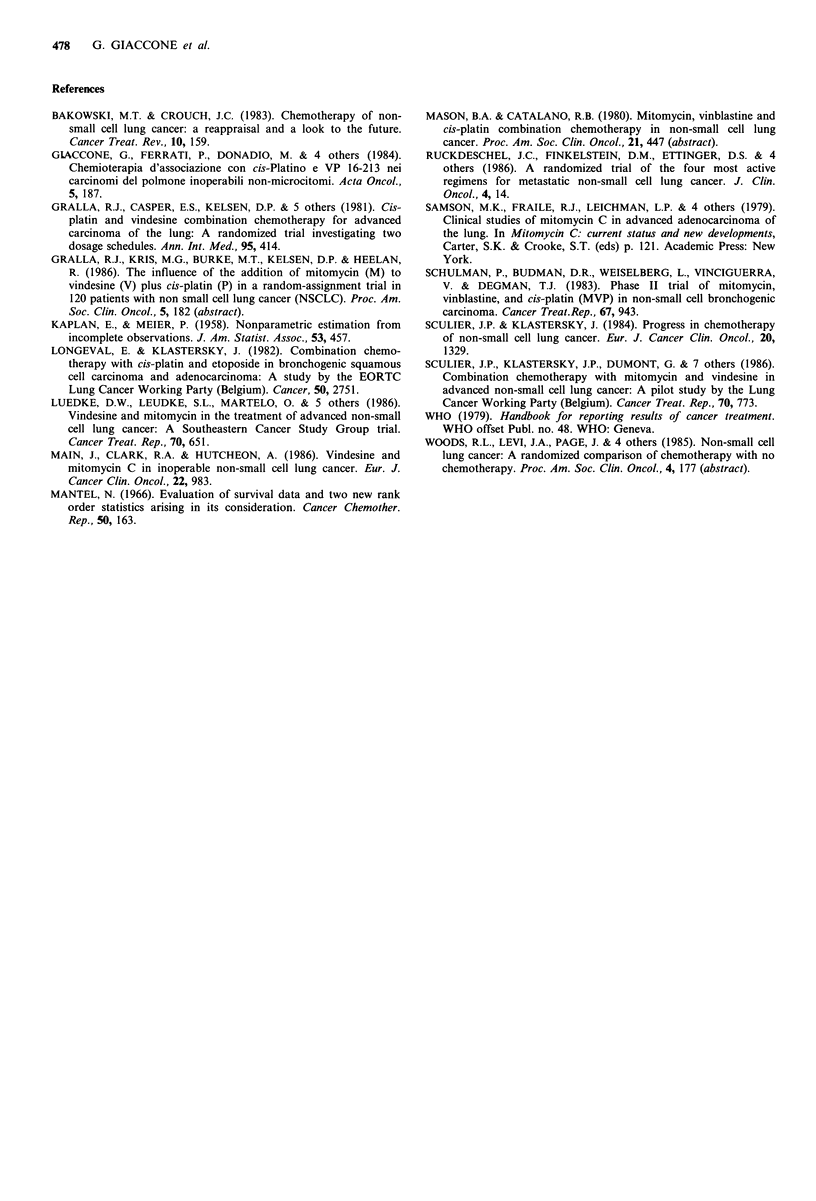


## References

[OCR_00451] Bakowski M. T., Crouch J. C. (1983). Chemotherapy of non-small cell lung cancer: a reappraisal and a look to the future.. Cancer Treat Rev.

[OCR_00462] Gralla R. J., Casper E. S., Kelsen D. P., Braun D. W., Dukeman M. E., Martini N., Young C. W., Golbey R. B. (1981). Cisplatin and vindesine combination chemotherapy for advanced carcinoma of the lung: A randomized trial investigating two dosage schedules.. Ann Intern Med.

[OCR_00479] Longeval E., Klastersky J. (1982). Combination chemotherapy with cisplatin and etoposide in bronchogenic squamous cell carcinoma and adenocarcinoma. A study by the EORTC lung cancer working party (Belgium).. Cancer.

[OCR_00485] Luedke D. W., Luedke S. L., Martelo O., Quesenberry P., Birch R., Schlueter J., Hake J., Logan T. (1986). Vindesine and mitomycin in the treatment of advanced non-small cell lung cancer: a Southeastern Cancer Study Group Trial.. Cancer Treat Rep.

[OCR_00491] Main J., Clark R. A., Hutcheon A. (1986). Vindesine and mitomycin C in inoperable non-small cell lung cancer.. Eur J Cancer Clin Oncol.

[OCR_00496] Mantel N. (1966). Evaluation of survival data and two new rank order statistics arising in its consideration.. Cancer Chemother Rep.

[OCR_00519] Schulman P., Budman D. R., Weiselberg L., Vinciguerra V., Degnan T. J. (1983). Phase II trial of mitomycin, vinblastine, and cisplatin (MVP) in non-small cell bronchogenic carcinoma.. Cancer Treat Rep.

[OCR_00530] Sculier J. P., Klastersky J., Dumont J. P., Vandermoten G., Rocmans P., Libert P., Ravez P., Becquart D., Mommen P., Dalesio O. (1986). Combination chemotherapy with mitomycin and vindesine in advanced non-small cell lung cancer: a pilot study by the Lung Cancer Working Party (Belgium).. Cancer Treat Rep.

[OCR_00525] Sculier J. P., Klastersky J. (1984). Progress in chemotherapy of non-small cell lung cancer.. Eur J Cancer Clin Oncol.

